# Preparation of hydrogel microsphere and its application in articular cartilage injury

**DOI:** 10.1016/j.mtbio.2025.101641

**Published:** 2025-03-08

**Authors:** Zehua Wang, Xiaoxia Li, Yaping Jiang, Tingyu Wu, Sijia Guo, Tao Li

**Affiliations:** a Department of Orthopaedic Surgery, The Affiliated Hospital of Qingdao University, Qingdao, 266003, China; b Department of Genetics and Cell Biology, School of Basic Medicine, Qingdao University, Qingdao, 266000, China; c Department of Oral Implantology, The Affiliated Hospital of Qingdao University, Qingdao, 266003, China

**Keywords:** Hydrogel microsphere, Articular cartilage injury, Biomaterial, Delivery platforms, Osteoarthritis, Tissue engineering, Anti-inflammatory properties, Stem cell recruitment

## Abstract

In recent years, hydrogel microspheres have garnered significant attention due to their unique structure and functionality, demonstrating substantial potential in articular cartilage injury repair. This paper provides a comprehensive overview of current strategies for cartilage injury repair and summarizes the materials and preparation methods of hydrogel microspheres. Furthermore, it highlights the multiple roles of hydrogel microspheres in cartilage repair, including inflammation control, regulation of chondrocyte metabolism, drug and cell delivery, lubrication improvement, and recruitment of endogenous stem cells. Finally, the paper discusses the application prospects of hydrogel microspheres, identifies current limitations and challenges, and offers insights to guide future research and practical applications in cartilage injury repair.

## Highlights

• Hydrogel microspheres in the repair of articular cartilage injuries are summarized.
• Various methods for the preparation of hydrogel microspheres are described.
• The role played by hydrogel microspheres in articular cartilage damage is summarized.

## 1 Introduction

Cartilage is a smooth, resilient, and flexible connective tissue layer covering the joint surfaces of limbs, primarily chondrocytes, cartilage matrix, and cartilage membrane. It facilitates frictionless joint movement and cushions against load-induced stress [1]. Due to the absence of direct blood and lymphatic supply in cartilage and chondrocytes being highly differentiated cells with poor proliferation and migration ability [2,3], cartilage exhibits reduced self-repair abilities, particularly with aging, making it highly susceptible to damage. Such damage frequently leads to degenerative joint conditions, notably osteoarthritis (OA), with the knee being the most affected joint, followed by the hip and ankle [4,5]. Currently, over 30 % of the global population experiences diseases linked to articular cartilage injury, with an increasing incidence rate that significantly impacts patient quality of life and imposes substantial social and economic burdens [6,7]. While clinical strategies for repairing articular cartilage injury—such as microfracture, subchondral bone drilling, osteochondral transplantation, and cell transplantation—are available, challenges persist. These include poor mechanical properties of newly formed fibrocartilage, limited donor sources, donor site complications, immune rejection of grafts, and inadequate integration with the host tissue [8]. Consequently, developing a cost-effective treatment strategy for cartilage injuries could yield significant economic and social benefits globally.

Cartilage repair strategies based on tissue engineering and regenerative medicine have gained significant attention in recent years. The development and application of biomimetic repair materials have made notable progress in replicating the structure and function of natural cartilage, offering new approaches for cartilage injury repair[[9], [10], [11]]. Hydrogels, composed of hydrophilic polymer chains forming a three-dimensional network, can absorb and retain large amounts of water while maintaining their structure and morphology [12,13]. They exhibit tunable physical, chemical, and mechanical properties, excellent biocompatibility, and responsiveness to external stimuli[[14], [15], [16]]. These attributes have enabled hydrogels to be widely applied in drug delivery, soft tissue filling, cell and molecule separation, artificial organs and prostheses, and biomaterial repair[14,[17], [18], [19]]. However, their clinical application is constrained by the high toxicity of cross-linking agents, limited mechanical properties, and poor stability in drug delivery systems [20,21]. Hydrogel microspheres are a specialized form of hydrogel, ranging from the micrometer to millimeter scale, and share fundamental properties with conventional hydrogels. Their small size enables *in vitro* preparation and crosslinking, followed by *in vivo* injection via a syringe [22]. This characteristic provides significant advantages over hydrogels crosslinked *in vivo*, as hydrogel microspheres exhibit greater crosslinking stability. Consequently, injectable suspensions containing these microspheres maintain better fluidity and prolonged retention *in vivo*, making them more suitable for injectable therapies across various tissues [23]. Furthermore, since hydrogel microspheres can be entirely prepared and crosslinked *in vitro*, they can be precisely modified and processed to meet specific requirements, enabling the fabrication of complex structures with multiple functionalities [24,25]. Their miniaturized size and morphology also contribute to a characteristic porous structure [22,26]. This structure enhances the specific surface area, mechanical strength, stability, and ductility while providing a scaffold for cell attachment, proliferation, and migration, thereby promoting tissue formation and growth [27]. Additionally, the porous architecture facilitates the free exchange of oxygen and nutrients between cells and the extracellular environment, enhancing cell activity and function [23]. Moreover, numerous studies demonstrate that hydrogel microspheres can be loaded with drugs [24,28,29], growth factors [25], or other biologically active substances [30], enabling controlled release and continuous delivery of repair-promoting factors [31,32]. The unique properties of hydrogel microspheres, including excellent biocompatibility, high water content, three-dimensional porous structure, controlled delivery of therapeutic agents, and injectability, make them ideal for creating a growth environment for chondrocytes. These features highlight their potential for promoting cartilage regeneration and repair, positioning hydrogel microspheres as a promising solution for future applications in this field.

**Fig. 1:**
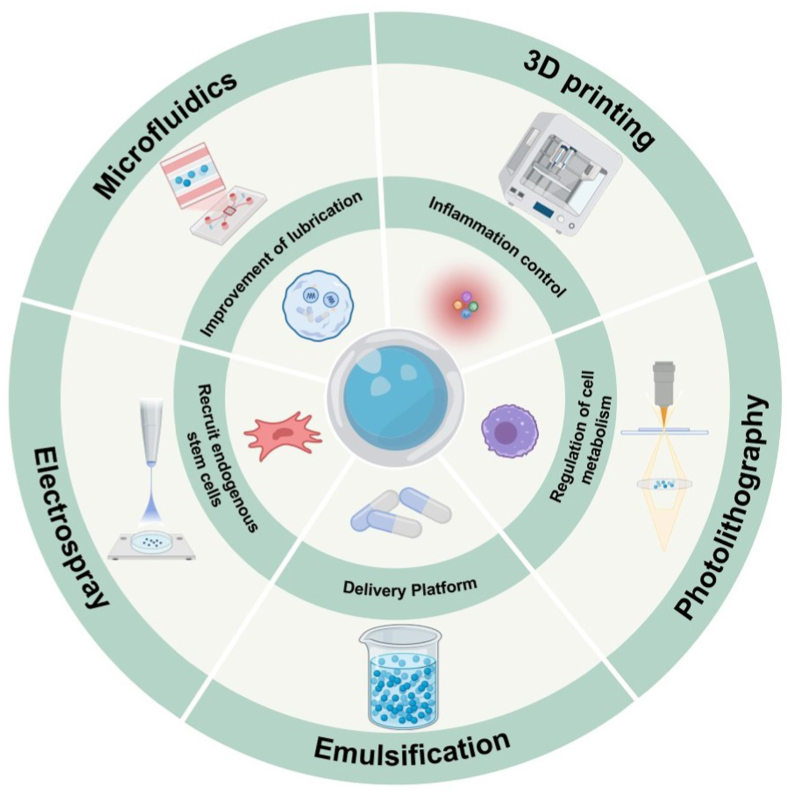
Preparation of hydrogel microspheres and their role in the repair of articular cartilage injury.

This paper reviews current biological strategies for promoting articular cartilage repair and summarizes the materials and preparation methods of hydrogel microspheres, including microfluidics, emulsification, electrospray, photolithography, and 3D printing. Additionally, it examines the diverse roles and mechanisms of hydrogel microspheres in articular cartilage repair. Finally, the paper discusses the future development and application prospects of functional hydrogel microspheres, aiming to inform subsequent research on their preparation and utilization in cartilage injury repair (Fig. 1).

## 2 Composition, structure, and function of articular cartilage

Articular cartilage is a specialized connective tissue with a high degree of structural integrity, consisting mainly of a small number of chondrocytes and a large amount of extracellular matrix (ECM) [33,34]. Chondrocytes, the sole cellular component of articular cartilage, are dispersed within the cartilage matrix and are responsible for synthesizing and maintaining its components [35]. The extracellular matrix predominantly comprises 70 % water and 30 % proteoglycans and fibrous elements, which possess water-retaining properties [36]. Collagen, the major fibrous component of the extracellular matrix, is essential for providing tensile strength. In mature cartilage, type II collagen is the most abundant, arranged in an interwoven pattern that imparts strength and toughness while playing a crucial role in chondrocyte mechanotransduction and maintaining cartilage elasticity [37,38]. Non-collagenous components, such as proteoglycan aggregates, contain negatively charged glycosaminoglycan chains that attract water molecules, conferring elasticity and resistance to compression on the cartilage [39]. The extracellular matrix components form a dynamic regulatory network that supports cellular behavior and preserves tissue homeostasis [34,40,41].

**Fig. 2:**
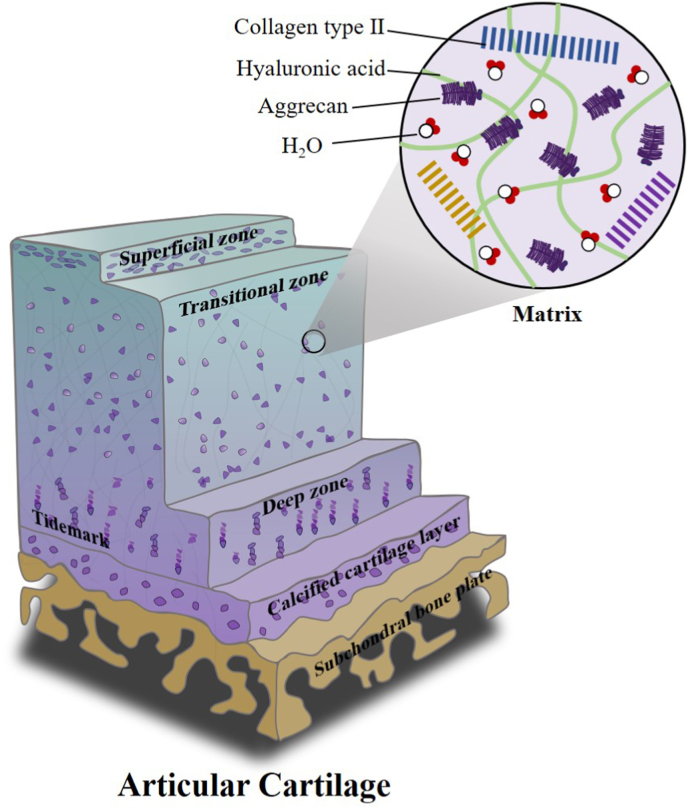
The architecture of osteochondral units and the schematic diagram of their constituents. Reproduced with permission.

Based on its composition, structure, and the morphology and function of chondrocytes, articular cartilage is typically divided into four distinct zones, progressing vertically from the cartilage surface: superficial, transitional, radial, and calcified cartilage layers [[42], [43], [44]]. Each layer has a unique structure and composition, including differences in chondrocyte morphology and volume, collagen fiber thickness and orientation, proteoglycan concentration, and water content adapted to their specific functional roles [45]. The superficial layer contains flattened, elliptical chondrocytes with long axes parallel to the articular surface. This layer is covered by a thin synovial membrane, providing a low-friction sliding surface for joint movement [44]. The chondrocytes in this layer secrete high levels of collagen and low levels of proteoglycans, resulting in the highest water content among the layers. The parallel arrangement of collagen fibers enhances resistance to tensile and shear stresses [46] and protects cartilage and synovial tissues from immune system invasion [47]. The transitional layer, thicker than the superficial layer, is characterized by spherical chondrocytes and randomly oriented collagen fibers, which mediate compatibility between the shear forces of the superficial layer and the compressive forces of the radial layer [44]. The radial layer contains spherical chondrocytes, thick collagen fibers, and abundant proteoglycans [48]. The collagen fibers in this layer exhibit a distinctive anisotropic structure, radially arranged from the calcified zone upward and perpendicular to the articular surface [49]. These features are critical for maintaining the mechanical stability and matrix balance of articular cartilage [50]. The calcified cartilage layer, the thinnest zone, connects to the subchondral bone and bridges hyaline cartilage and bone tissue. This layer prevents the invasion of blood vessels and other tissues into the cartilage, thereby preserving the functional integrity and independence of articular cartilage (Fig. 2).

## 3 Types of articular cartilage injuries and treatment strategies

### 3.1 Types of articular cartilage damage

Cartilage tissue primarily consists of chondrocytes and an extracellular matrix, with collagen as the main component of the matrix. Collagen forms the basic three-dimensional cartilage structure through its ordered arrangement [44,51,52]. Under normal conditions, joint stress induces fluid movement within the articular cartilage matrix, distributing the load across the intrachondral and subchondral bone [14,53]. However, excessive or rapid loading disrupts the macromolecular framework of the matrix, causing articular cartilage damage [54]. Chondrocyte apoptosis follows, driven by oxidative stress, inflammation, and enzyme-mediated degradation, disrupting the structure and composition of the extracellular matrix. This ultimately leads to the destruction and loss of cartilage tissue integrity [[55], [56], [57]]. Cartilage damage can be classified into two types based on severity [58,59]. The first involves alterations to the cartilage matrix without chondrocyte damage. In this case, chondrocytes can sense changes in the matrix and synthesize new molecules to repair it. However, if matrix damage exceeds the repair capacity of chondrocytes, excessive loading leads to cartilage degeneration [60]. The second type involves simultaneous damage to both the extracellular matrix and chondrocytes. Depending on the depth of the damage, this can be further classified as cartilage damage or osteochondral damage. Cartilage damage is confined to the cartilage layer, while osteochondral damage penetrates the subchondral bone, causing bleeding and triggering an inflammatory response [61,62].

### 3.2 Therapeutic strategies for articular cartilage injuries

Articular cartilage lacks nutrient-supporting tissues such as blood vessels, lymphatics, and nerves, making self-repair after injury challenging [63]. The depth, mechanisms, and pathogenesis of cartilage defects vary between injuries, necessitating tailored treatment strategies. Current clinical treatments for articular cartilage injuries primarily include Subchondral drilling, osteochondral transplantation, cell transplantation, and cartilage tissue engineering (Table 1)[[64], [65], [66]].

**Table 1:** Advantages and disadvantages of therapeutic strategies for articular cartilage injuries.

Method	Advantages	Disadvantages	References
Subchondral drilling	Induce bone marrow hemorrhage to promote the formation of new cartilage tissue, including some hyaline cartilage.	Filled fibrocartilage lacks biomechanical properties and viscoelasticity; the repair tissue is mostly fibrocartilage	[69]
Osteochondral transplantation	Autologous osteochondral grafts offer superior mechanical stability and biocompatibility, while allogeneic grafts enable the repair of larger damaged areas.	Limited autologous donor area, affecting the function of the non-weight-bearing area; risk of immune rejection with allografts; limited availability and quality of grafts	[74,75]
Cell transplantation	Fully differentiated chondrocytes provide faster repair, and undifferentiated MSCs provide additional cell sources	Autologous chondrocytes are from limited sources and, in most cases, are difficult to be cultured on a large scale *in vitro*	[79]
Periosteal or Perichondrial Grafting	The germinal layer provides a supportive environment for chondrocyte growth and a certain number of mesenchymal stem cells	The newly formed cartilage has poor mechanical properties and is prone to fibrotic degeneration	[14,80]
Cartilage tissue engineering	The scaffolds mimic cartilage properties, are loaded with bioactive factors that promote repair, and can be optimized for performance in composite scaffolds and composite hydrogels.	The complexity of manufacturing scaffolds, lack of cytokine stability, and difficulty in controlling the release	[87,90,95]

#### 3.2.1 Subchondral drilling

Osteochondral joint injuries involving the subchondral bone can trigger the repair of the damaged area through hemorrhage and an inflammatory response. In some cases, the repaired tissue demonstrates the potential to compensate for articular cartilage function. Clinically, procedures aim to induce bone marrow hemorrhage and promote new cartilage formation by arthroscopically grinding and removing the superficial cartilage layer with a high-speed drill and drilling into the subchondral bone [67,68]. However, the resulting repair tissue contains some hyaline cartilage but is predominantly fibrous tissue. Consequently, most repair tissues deteriorate to varying degrees after maintaining compensatory function for a while. Knutsen et al. examined pathological sections from over 20 patients who underwent microfracture treatment of subchondral bone and found that hyaline cartilage comprised only 11.4 % of the repair tissue. In contrast, 17.1 % comprised a mixed hyaline cartilage and fibrocartilage structure. The remaining tissue was primarily fibrous cartilage [69]. Furthermore, studies have shown that combined osteochondral injuries often achieve more satisfactory bony repair at the bone defect site, while cartilage injuries typically result in fibrocartilage repair [70]. This discrepancy is likely related to the biological and mechanical differences between the articular and bone marrow cavities. The differential differentiation observed in these environments suggests the potential for introducing undifferentiated cells to direct the cartilage repair response and reconstruct the articular surface. However, poor long-term repair outcomes, the lack of biomechanical properties and viscoelasticity of fibrocartilage, and its unsuitability for geriatric patients limit its clinical application.

#### 3.2.2 Osteochondral transplantation

Osteochondral transplantation involves transferring intact columnar or block grafts containing cartilage and subchondral bone from donor to recipient sites to repair articular cartilage defects [71,72]. Based on the graft source, osteochondral grafts are categorized as autologous or allogeneic. Unlike fibrocartilage repairs generated through microfracture techniques, this method introduces a new hyaline cartilage surface. Autologous osteochondral grafts, typically harvested from the patient's non-weight-bearing areas, provide superior mechanical stability and biocompatibility. However, the availability of donor tissue is limited, and harvesting may impair the function of the donor site [73]. In contrast, allogeneic grafts, often sourced from cadavers, can repair larger injuries but are associated with risks of immune rejection, as well as limited availability and inconsistent quality [74]. Granulated articular cartilage implantation, derived from osteochondral grafting, involves pulverizing donor cartilage into 1–2 mm^2^ granules implanted into the defect site. This approach reduces the amount of donor cartilage required and minimizes damage to the donor site. However, transplanted cartilage particles remain difficult to integrate with surrounding cartilage tissue, failing to address the fundamental challenges of osteochondral transplantation [75].

#### 3.2.3 Cell transplantation

Cell transplantation is an effective method for repairing articular cartilage defects [76]. This technique involves culturing and expanding the patient's chondrocytes, which are then transplanted into the cartilage defect site to promote cartilage formation and regeneration. Research has increasingly focused on using fully differentiated chondrocytes and undifferentiated mesenchymal stem cells (MSCs) [53]. Fully differentiated chondrocytes possess well-defined chondrocyte properties, enabling them to rapidly exert reparative effects and directly participate in new cartilage formation after transplantation [77]. In contrast, undifferentiated MSCs can differentiate into chondrocytes under suitable conditions, providing an additional cell source for cartilage repair [78]. Chondrocytes are typically harvested from the non-weight-bearing area of the patient's joint surface. After *in vitro* culture and expansion, the cells are transplanted to the defect site and covered with a chondrocyte membrane or fascia to prevent cell loss. However, the limited availability of autologous chondrocytes and the challenges of large-scale *in vitro* chondrocyte culture significantly restrict the widespread application of this approach [79].

#### 3.2.4 Periosteal or Perichondrial grafting

Periosteal or chondrocyte membrane transplantation is a technique used to promote cartilage regeneration by transplanting periosteum or chondrocyte membranes to the injured area. Adult periosteum and chondrocyte-derived membranes contain many MSCs, exhibiting strong multidirectional differentiation potential and biological activity [80]. Transplanted periosteum and chondrocyte membranes provide a supportive environment for chondrocyte growth and supply MSCs. Under appropriate conditions, these cells differentiate into chondrocytes and form new cartilage tissue through continuous proliferation, thereby repairing damaged articular cartilage [81]. Clinical studies have shown that patients with articular cartilage injuries who undergo periosteal or chondrocyte membrane transplantation experience significant short-term symptom relief, including reduced pain. However, the newly formed cartilage tissue struggles to withstand the long-term mechanical demands of weight-bearing joints and fails to achieve satisfactory mechanical properties [14]. Over time, the new cartilage tissue undergoes fibrotic degeneration, eventually leading to the recurrence of OA [14].

#### 3.2.5 Cartilage tissue engineering

Advances in biological and engineering technologies have enabled cartilage regeneration based on tissue engineering principles. Cartilage tissue engineering comprises three main components: scaffolds, seed cells, and growth factors. Biocompatible scaffolds with functional properties not only provide an optimal living environment for seed cells but also facilitate the loading and release of growth factors, promoting intercellular interactions to induce the formation of target cells and tissues [82]. The structure and materials of scaffolds mimic the mechanical and biological properties of natural cartilage, supporting cell attachment, growth, and differentiation while controlling growth factor release and allowing gradual replacement by regenerated tissues [83]. This approach offers innovative solutions for repairing articular cartilage damage. However, replicating the complex morphology and structure of natural cartilage remains challenging due to its varied chemical and mechanical properties at different layers [84]. Researchers have developed composite scaffolds with multilayered designs that mimic the cartilage's hierarchical structure, providing targeted mechanical and biological support to enhance cartilage regeneration [85]. Zhou et al. designed a heterogeneous three-layer scaffold mimicking the biochemical composition and structure of hyaline cartilage, calcified cartilage, and subchondral bone, incorporating type I collagen, hyaluronic acid, and nano-hydroxyapatite. The scaffold exhibited high porosity, effectively promoting cell adhesion and proliferation. Even after 30 days of degradation, 50%–75 % of its structure was retained, supporting osteochondral tissue regeneration [86]. Similarly, Liu et al. developed a 3D bioprinted multilayered scaffold containing bone marrow mesenchymal stem cells (BMSCs) to repair articular cartilage injuries in an osteoarthritis rat model. The scaffold supported BMSC growth and proliferation, facilitated the production of chondrocyte-specific extracellular matrix, and enhanced type II collagen synthesis. Additionally, it inhibited the expression of inflammatory factors like interleukin-1β, significantly improving joint function in the affected limb [87].

Synthetic and natural biomaterials each offer distinct advantages and limitations. Natural polymers exhibit excellent biocompatibility but have poor mechanical properties [88]. In contrast, synthetic polymers provide more controllable chemical modifications and cross-linking densities, enabling them to mimic better the mechanical properties of articular cartilage [89]. Consequently, increasing research efforts have focused on developing natural-synthetic polymer composite hydrogels to optimize mechanical properties and biological functions for enhanced articular cartilage repair. For example, Chen et al. designed a three-dimensional scaffold combining decellularized cartilage extracellular matrix with waterborne polyurethane (WPU). The addition of ECM significantly improved cell adhesion and proliferation. Furthermore, the WPU-ECM scaffold, when combined with microfracture surgery, successfully induced hyaline cartilage regeneration *in vivo* [90].

Cytokines play a vital role in articular cartilage formation and the maintenance of normal function. As key signaling molecules, they regulate cell behavior, influencing proliferation, differentiation, migration, and extracellular matrix formation [91]. Numerous studies have demonstrated that loading bioactive factors into scaffold materials can enhance chondrocyte repair and regeneration [25,92]. However, the stability of these factors within scaffolds is often insufficient, and their rapid release limits therapeutic efficacy. To address this, slow-release technology has been introduced [93,94]. Chen et al. developed a stromal-derived factor-1α (SDF-1α)/transforming growth factor-β1 (TGF-β1)-loaded silk fibroin-porous gelatin scaffold. This scaffold provided sustained release of these factors, enhancing cartilage repair by promoting the homing, migration, and chondrogenic differentiation of MSCs. In a rat knee joint osteochondral defect model, the scaffold effectively promoted cartilage regeneration and achieved significant defect repair 12 weeks after transplantation [95].

## 4 Materials of hydrogel microspheres

To replicate the composition and structure of natural cartilage tissue, most materials used for cartilage regeneration are organic compounds, including natural and synthetic polymers that mimic the extracellular matrix [96]. The selection of biomaterials is critical for hydrogel microsphere fabrication, as their composition and surface chemical groups influence cell adhesion, morphology, and overall tissue repair efficacy [97]. Common natural biomaterials for hydrogel microsphere preparation include gelatin, hyaluronic acid (HA), alginate, and chitosan. Additionally, synthetic polymers such as polyethylene glycol (PEG), polylactic-co-glycolic acid (PLGA), and methacrylate gelatin (GelMA) can be chemically processed or polymerized with other substances, making them widely utilized in hydrogel microsphere fabrication.

### 4.1 Natural polymers

#### 4.1.1 Gelatine

Gelatin, a natural polymer derived from collagen through irreversible thermal denaturation or hydrolysis, exhibits properties dependent on collagen type, source, and denaturation process [98]. Compared to collagen, gelatin has lower antigenicity and better biocompatibility, reducing immune responses associated with prolonged presence *in vivo*. Furthermore, gelatin contains arginine-glycyl-aspartic acid (RGD) sequences and integrative molecules that enhance binding to surrounding tissues and cells, promoting chondrocyte proliferation and differentiation [99,100]. The mechanical properties and *in vivo* stability of gelatin can be improved through artificial crosslinking and structural modulation [101], making gelatin-based hydrogel microspheres widely used in cartilage tissue engineering.

#### 4.1.2 Hyaluronic acid

Hyaluronic acid, a naturally occurring glycosaminoglycan, consists of repeating disaccharide units of N-acetyl-d-glucosamine and d-gluconic acid [102]. It interacts with extracellular molecules to form a complex matrix and regulates cell growth, migration, and differentiation [103]. Cellular responses to hyaluronic acid depend on its chain length, molecular weight, synthesis conditions, and receptor expression [104]. High-molecular-weight hyaluronic acid exhibits immunosuppressive and antiangiogenic properties, reduces inflammation, and promotes wound healing while preventing scar formation. Conversely, low-molecular-weight hyaluronic acid can stimulate inflammatory cytokine secretion through alternative receptor interactions[[105], [106], [107]]. Sulfated hyaluronic acid serves as a potential anabolic agent for tendons and cartilage by recruiting mesenchymal stem cells to injury sites and promoting cell differentiation [108]. Disulfide crosslinked hyaluronic acid hydrogels exhibit excellent biocompatibility, resistance to enzymatic degradation, and promotion of fibroblast adhesion and proliferation, making them ideal for extracellular matrix construction [109]. These properties highlight the significant potential of hyaluronic acid in cartilage repair and regeneration.

#### 4.1.3 Alginate

Alginate, a natural anionic polysaccharide derived from algae, primarily consists of β-D-mannose and α-L-gulose units and is widely used for drug release and cell delivery [110,111]. Alginate undergoes physical crosslinking with divalent cations to form stable hydrogels [112]. Calcium ions, abundant in joint cavities, are the most commonly used crosslinking agents in cartilage tissue engineering [113]. Thomas et al. developed calcium alginate Janus microspheres by crosslinking a 4 % sodium alginate solution with 2.5 % calcium chloride, subsequently loading them with mesenchymal stem cells for cartilage repair and regeneration [114]. However, alginate hydrogel microspheres exhibit limited protein adsorption and cell adhesion due to their high water content and lack of binding sites.

#### 4.1.4 Chitosan

Chitosan, derived from chitin via deacetylation, possesses N-acetylglucosamine moieties similar to those found in articular cartilage, providing specific binding sites for cytokines and adhesion proteins [115]. However, its poor solubility in water and most organic solvents restricts its applications [116]. To overcome this limitation, researchers have chemically modified its reactive groups, yielding derivatives with improved biocompatibility and degradability while retaining its essential properties [117,118]. Kim et al. utilized an emulsification method to prepare chitosan microspheres containing TGF-β1, where the porous structure facilitated chondrocyte proliferation and enhanced cartilage repair [119].

### 4.2 Polyethylene glycol

#### 4.2.1 Polyethylene glycol

PEG is a polymer synthesized via ethylene oxide polymerization with water or ethylene glycol. It exhibits low toxicity, low immunogenicity, and biodegradability, with degradation products excreted through the kidneys, making it highly suitable for joint injury repair [120]. Yao et al. developed gelatin-based PEG composite microspheres using microfluidics, incorporating a dual slow-release system of liposomes and hydrogels to extend Kartogenin retention in the joint cavity, thereby promoting mesenchymal stem cell differentiation into chondrocytes and accelerating cartilage regeneration. Fluorescent signals enabled real-time monitoring of the repair process [121].

#### 4.2.2 Poly(lactic acid-co-glycolic acid)

PLGA, a widely used biodegradable polymer synthesized via polycondensation of lactic and glycolic acids, offers excellent biocompatibility, biodegradability, and tunable physicochemical properties, making it suitable for drug delivery, tissue engineering, and cartilage repair [122,123]. PLGA-based composite scaffolds incorporating bioactive molecules provide structural support for chondrocytes and enhance cartilage regeneration. For instance, composite scaffolds composed of PLGA and hyaluronic acid methacrylate, featuring a radial pore structure, reduced inflammatory factor expression and promoted the regeneration of cartilage and subchondral bone, demonstrating strong potential for in situ cartilage repair [124].

#### 4.2.3 Gelatin methacryloyl

GelMA is a gelatin derivative modified with methacrylate groups, that retains essential cell adhesion sequences such as RGD peptides, enabling it to mimic the cartilage extracellular matrix microenvironment and support chondrocyte proliferation and differentiation [125]. Its three-dimensional porous structure facilitates cell migration and nutrient exchange. For example, GelMA/PSBMA microspheres (G/S HMS) fabricated via microfluidic technology exhibit a smooth, dense structure that maintains its spherical shape under friction, providing rolling lubrication. Additionally, anti-type I collagen antibodies incorporated onto the microsphere surface selectively bind to type I collagen at injury sites, enabling precise lubrication and repair [126].

In summary, selecting hydrogel microsphere materials for cartilage repair requires consideration of biocompatibility, degradability, mechanical properties, cellular support, and drug or growth factor delivery capabilities to ensure optimal tissue regeneration. Natural polymers offer excellent biocompatibility and support for cell attachment and growth, closely resembling cartilage tissue. However, their structural consistency during production can be challenging to maintain. Conversely, synthetic polymers provide greater structural consistency but often exhibit lower biocompatibility. Thus, recent research has focused on hybrid hydrogels combining natural and synthetic polymers to optimize mechanical properties, degradation rates, and biocompatibility, ultimately enhancing cartilage repair outcomes.

## 5 Preparation of hydrogel microspheres

**Fig. 3:**
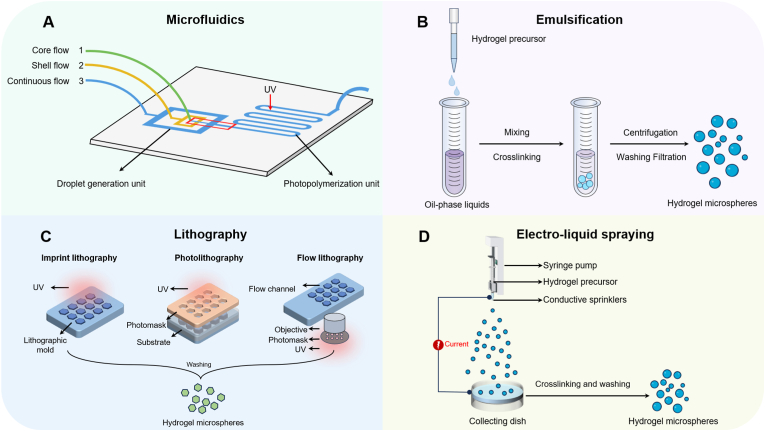
Schematic diagram of the hydrogel microsphere preparation method. (A) Microfluidics is the generation of monodisperse droplets and in situ cross-linking curing to form hydrogel microspheres with uniform particle size and controllable structure through precise shearing of two-phase fluids in a microscale channel. (B) Emulsification is the use of immiscible solutions mixed and dispersed to form an emulsion, which is then cured by a cross-linking reaction to form microspheres. (C) The electrospray method uses a high-voltage electric field to atomize a liquid and form tiny droplets that are subsequently cross-linked to form hydrogel microspheres. (D) Photolithography is used to obtain hydrogel microspheres of specific shapes and sizes by coating a photosensitive material on a substrate, exposing it using a photolithography mask and UV light, and subsequently removing the unexposed areas by a development process, which ultimately results in hydrogel microspheres of specific shapes and sizes.

Hydrogel microspheres are nanosized, spherical particles prepared from hydrophilic polymers through cross-linking [22]. Compared with traditional hydrogels, hydrogel microspheres retain the fundamental properties of hydrogels while offering the advantage of being injectable through small needles and catheters, enabling minimally invasive delivery of cells and biological agents for therapeutic applications [127]. Additionally, their porous structure promotes cell proliferation and differentiation and maintains a high survival rate for encapsulated cells, even under high cell-loading conditions[22,[128], [129], [130]]. The porous network of hydrogel microspheres allows for the encapsulation of drugs and bioactive molecules. By modulating the surface area, the release rate of loaded medications can be controlled, enabling sustained drug release and long-term therapeutic effects [24,25,29]. These unique properties have made hydrogel microspheres highly studied in drug delivery and regenerative medicine. Studies have explored various preparation techniques, highlighting how their *in vivo* performance is closely related to composition, structure, and processing methods [131]. Analyzing the advantages and limitations of different preparation techniques is essential for advancing research and broadening applications (Table 2). Currently, the primary methods for preparing hydrogel microspheres include microfluidics, emulsification, electrospray, photolithography, and 3D printing (Fig. 3).

**Table 2:** Advantages and disadvantages of preparation of hydrogel microspheres.

Method	Advantages	Disadvantages	References
Microfluidics	The microspheres are highly uniform in size and shape; Multifunctionality; precise control of reaction conditions, modulation of hydrogel physicochemical properties	High equipment maintenance costs; complex operation; high technical requirements for personnel; low efficiency of large-scale production	[133,134]
Emulsification	Precise control of microsphere size and structure; Easy operation; low cost; high yield; controllable	Uneven dispersion of emulsion; wide particle size distribution of microspheres; Complicated operating procedure, precise control of emulsification conditions required	[136]
Electrospray	Precise control of microsphere size; flexible material selection, and the ability to prepare microspheres with complex structures	Limited production capacity; high equipment requirements; sensitivity to the operating environment	[[142], [143], [144]]
Photolithography	Specific size and morphology of microspheres can be obtained, allowing direct preparation of cell-containing microspheres with reduced cellular damage	Relatively low production	[146,147]
3D printing	The diameter, shape, and internal structure of the microspheres can be precisely controlled; different materials can be mixed to build multifunctional microspheres	Complex equipment parameters, difficulty in maintaining biomolecular activity	[151,152]

### 5.1 Microfluidics

Microfluidics is a technique for manipulating and handling fluids at the micrometer or nanometer scale, capable of simultaneously regulating multiple fluid flow phases to produce nanoscale homogeneous hydrogel microspheres with predefined compositions and stable structures [15,132,133]. Microspheres prepared using microfluidics have garnered significant attention and are considered promising for biomedicine, tissue engineering, and regenerative medicine applications. Li et al. successfully prepared gelatin methacrylate microspheres using freeze-drying microfluidics combined with platelet-derived growth factor-bb and exogenous MSCs. These microspheres exhibited uniform and controllable sizes, elasticity, and biocompatibility. Ex vivo experiments demonstrated superior secretory properties and anti-inflammatory effects, with the microspheres enhancing the paracrine effect of MSCs and slowing OA progression [25]. Similarly, Xiao et al. developed immune cell-mobilized hydrogel microspheres using microfluidics, loading them with chemokines, macrophage antibodies, and engineered extracellular vesicles via covalent and non-covalent bonding. Experimental results revealed that these microspheres efficiently recruited, captured, and reprogrammed pro-inflammatory macrophages, improving the joint inflammatory microenvironment and slowing OA progression [134]. This method achieves high consistency in microsphere size and shape by precisely controlling fluid flow rates, channel structures, and fluid intersections. It also enables the rapid and efficient production of hydrogel microspheres through controlled fluid flow in microchannels [133]. The microfluidic system integrates multiple functional modules, such as cross-linking reactions and surface modifications, to provide multifunctionality for hydrogel microspheres. Additionally, it allows precise control of chemical concentrations, pH, and other reaction conditions during preparation, enabling fine-tuning of the hydrogels’ physical and chemical properties [135]. However, microfluidic devices are associated with high maintenance costs, complex operation processes, and technical demands for precise fluid control and stable environments. Furthermore, reliance on microchannel design and operation limits scalability for large-scale production.

### 5.2 Emulsification

Emulsification is a widely used method for preparing hydrogel microspheres. This technique involves dispersing one liquid into another immiscible liquid to form an emulsion, which is subsequently cured through cross-linking reactions to produce microspheres [131]. Based on the operational method, emulsification can be classified into single and double emulsification. Selecting the appropriate emulsification method according to the properties of the materials and drugs used ensures efficient drug loading, delivery, and release [136].

#### 5.2.1 Single emulsification

This method involves dispersing an aqueous solution of a water-soluble monomer or polymer into an oil phase using high-speed shear techniques to form an oil-in-water emulsion. Chemical or physical cross-linking is then used to solidify the hydrogel precursor in the aqueous phase, forming hydrogel microspheres [137]. Single emulsification is suitable for encapsulating hydrophobic drugs and is characterized by its simplicity, low material loss, higher yields, and controllability [138]. However, the instability of the water-oil interface often results in uneven emulsion dispersion, leading to a wider particle size distribution and making it difficult to achieve highly homogeneous microspheres.

#### 5.2.2 Double emulsification

This technique first creates a primary emulsion, either water-in-oil (W/O) or oil-in-water (O/W). The primary emulsion is then emulsified into a third immiscible liquid, forming a double emulsion such as water-in-oil-in-water (W/O/W) or oil-in-water-in-oil (O/W/O). The inner hydrogel precursor is subsequently cured through chemical or physical cross-linking to form microspheres [139]. Although double emulsification is more complex and requires precise control of emulsification conditions, it enables the preparation of hydrogel microspheres with intricate structures, making it advantageous for applications in drug-controlled release and cell encapsulation. By encapsulating active substances (e.g., drugs, proteins, or enzymes) in the inner aqueous phase, these microspheres can achieve functions such as sustained release and targeted delivery [140]. Additionally, this method allows for finer control over microsphere size and structure, resulting in improved uniformity [141].

### 5.3 Electrospray

Electrospray is a technique that utilizes a high-voltage electric field to atomize a liquid into tiny droplets. The process begins by ejecting a hydrogel precursor solution through a syringe with a strong electric field, which balances the liquid's surface tension. When the electric field force exceeds the liquid's surface tension, the liquid forms a conical structure (Taylor cone) at the nozzle, emitting fine droplets from its tip. These droplets are collected on a metal plate or in a conductive liquid and solidified through cross-linking reactions, forming hydrogel microspheres[[142], [143], [144]]. Liao et al. prepared alginate-gelatin hydrogel microspheres loaded with adipose-derived stem cells (ADSCs) using electrospray (Alg-Gel-ADSCs MSs) and evaluated their effects on articular cartilage defect repair in SD rats. Compared with conventional alginate microspheres, Alg-Gel-ADSCs MSs significantly improved the survival rate of ADSCs, promoted their proliferation and differentiation, and demonstrated superior cartilage repair [145]. By adjusting parameters such as voltage, flow rate, and nozzle diameter, the size of hydrogel microspheres can be precisely controlled, achieving uniform size and high encapsulation efficiency. Moreover, electrospray offers flexibility in material selection, accommodating various hydrogel precursor solutions and enabling the preparation of microspheres with complex structures. However, the technique has limitations, including low throughput, high equipment requirements, and sensitivity to the operating environment.

### 5.4 Lithography

Lithography is a method for preparing hydrogel microspheres and includes techniques such as imprint lithography, photolithography, and flow lithography. This process involves applying a photosensitive material to a substrate, exposing it to UV light through a photolithography mask, and removing the unexposed areas via a development process to produce hydrogel microspheres with specific shapes and sizes [146]. A key advantage of photolithography is its ability to produce microspheres with precise sizes and morphologies by tightly controlling the geometrical features of the mold [147]. Furthermore, Lithography allows for the direct preparation of hydrogel microspheres containing cells by precisely controlling the light-exposed area, eliminating the need for oil phases or surface activators and thereby reducing potential cell damage [146]. However, the rate of microsphere production is limited by the dimensions of the preparation molds, the size of the photolithography mask, and the field of view of the light source or objective, resulting in relatively low production yields.

### 5.5 3D printing

3D printing technology is renowned for its ability to produce high-precision, personalized hydrogel microspheres using computer-aided design (CAD). This technique enables precise control over the diameter, shape, and internal structure of microspheres to meet the requirements of specific applications, making it highly versatile for areas such as drug delivery and tissue engineering [148,149]. Furthermore, 3D printing allows the integration of different materials during the preparation process to create versatile microspheres. Bioactive molecules and cells can be combined with biomaterials to form biocarriers featuring open and porous 3D structures. These structures closely mimic natural biological tissues, enhancing their suitability for biomimetic designs in biomedical applications [150]. Compared with traditional preparation methods, 3D printing offers superior precision in controlling the diameter and structure of hydrogel microspheres. This results in a uniform pore distribution within the microspheres, enabling consistent drug release in all directions. Consequently, it increases local drug concentrations while reducing drug degradation and inactivation in the body [151]. Additionally, studies have demonstrated that hydrogel microspheres produced via 3D printing can effectively promote blood vessel and nerve growth, facilitating tissue repair and regeneration. These features make 3D printing highly promising for wound healing, tissue injury, and repair [152].

In recent years, 3D printing technology has been extensively utilized to prepare hydrogel microspheres, addressing the limitations of other preparation methods. Gefitinib (GEF), a small molecule drug used to treat cancer peritoneal metastasis and postoperative adhesions, faces challenges due to its high clearance rate, which hinders uniform and sustained drug distribution in the abdominal cavity. To overcome this, Remo Eugster et al. prepared multilayered vesicular alginate microspheres loaded with GEF using electromagnetic droplet nozzle printing. These microspheres enabled the sustained release of GEF *in vivo*, enhancing drug efficacy and prolonging its duration of action. This study highlights the potential of 3D-printed alginate microspheres containing liposomes for intraperitoneal drug delivery, offering innovative approaches for treating cancer peritoneal metastasis and postoperative adhesions [153]. Similarly, Wen et al. utilized 3D bioprinting to develop a 3D-G-CSF-SRM system by integrating granulocyte colony-stimulating factor (G-CSF) with slow-release microspheres (SRM). This system allows for individualized spatial control of drug distribution and structure, enabling G-CSF to remain in the body for extended periods and release gradually, thereby improving the precision and efficiency of treatment [154]. 3D printing technology can also be combined with other techniques to prepare hydrogel microspheres. Pan et al. designed a modular hydrogel bioink containing chondrocyte-embedded microspheres for 3D printing of multiscale composite scaffolds to support cartilage repair. They prepared gelatin methacrylate/alginate microspheres using microfluidics, which preserved the viability of embedded chondrocytes after cryo-resuscitation. The bioink facilitated the creation of hydrogel-based multiscale scaffolds through 3D printing. Cells cultured in these scaffolds exhibited robust proliferation, differentiation, and good biocompatibility *in vivo* [155]. Additionally, 3D printing devices play a critical role in regulating the size of hydrogel microspheres. A recent study developed a size-adjustable flow-focused droplet microdevice using 3D printing technology, enabling precise and rapid droplet size and generation frequency control. Using this device, researchers successfully synthesized a range of Calcium alginate microspheres containing alveolar epithelial basal cells [156]. The microspheres’ varying sizes provide diverse cell-encapsulation models for drug screening, facilitating the study of drug effects in different environments and offering a novel tool for personalized therapy and drug development.

## 6 Application of hydrogel microspheres in cartilage injury repair

Articular cartilage is a non-vascular, non-innervated connective tissue with slow cellular metabolism, which limits its capacity for self-repair and regeneration following injury [33,34,157]. Untreated cartilage defects can progressively extend into surrounding healthy cartilage, leading to further degeneration, wear, and potentially osteoarthritis [158,159]. Thus, exploring innovative strategies for cartilage regeneration is of significant clinical importance. Traditionally, treatments for articular cartilage injuries have included medication, physical therapy, and surgical interventions. While these approaches can temporarily alleviate pain and partially restore cartilage function, long-term clinical observations indicate that their effectiveness remains unsatisfactory [160].

Hydrogel microspheres, a class of polymeric materials with a three-dimensional mesh structure, have garnered significant attention due to their structural similarity to the extracellular matrix [15,121]. This structure supports cell adhesion and growth before injection while promoting cell migration, proliferation, and the exchange of nutrients and metabolites through the interstitial spaces, thereby accelerating new tissue formation [15,27]. Furthermore, functionalized designs can enhance the delivery of bioactive molecules and cells, amplifying their therapeutic potential [30,161,162]. Consequently, hydrogel microspheres offer substantial advantages in promoting tissue regeneration and improving repair outcomes. This section reviews the roles of hydrogel microspheres in cartilage regeneration, including controlling inflammation, modulating immune responses, regulating chondrocyte metabolism, serving as delivery platforms, improving lubrication, and recruiting endogenous stem cells.

### 6.1 Inflammation control and immune regulation

The progression of cartilage damage is primarily driven by inflammatory responses and immune mechanisms [163]. Initial mechanical injuries or pathological factors activate the immune system, with pro-inflammatory cytokines such as interleukin-1 β (IL-1β) and tumor necrosis factor-α triggering inflammatory responses. These responses recruit immune cells, including macrophages, and promote the release of inflammatory mediators and matrix metalloproteinases (MMPs), accelerating cartilage matrix degradation[[164], [165], [166]]. Concurrently, oxidative inflammation-related stress increases reactive oxygen species (ROS) levels, damaging chondrocytes and activating signaling pathways like NF-κB and MAPK. These pathways further upregulate inflammatory gene expression, perpetuating the inflammatory cycle [15,167]. Persistent inflammation exacerbates synovitis and immune dysregulation, creating a vicious cycle leading to increased chondrocyte apoptosis, impaired regeneration, progressive cartilage degradation, and functional loss [167]. Effective inflammation modulation and ROS production reduction are crucial for promoting cartilage repair and regeneration.

**Fig. 4:**
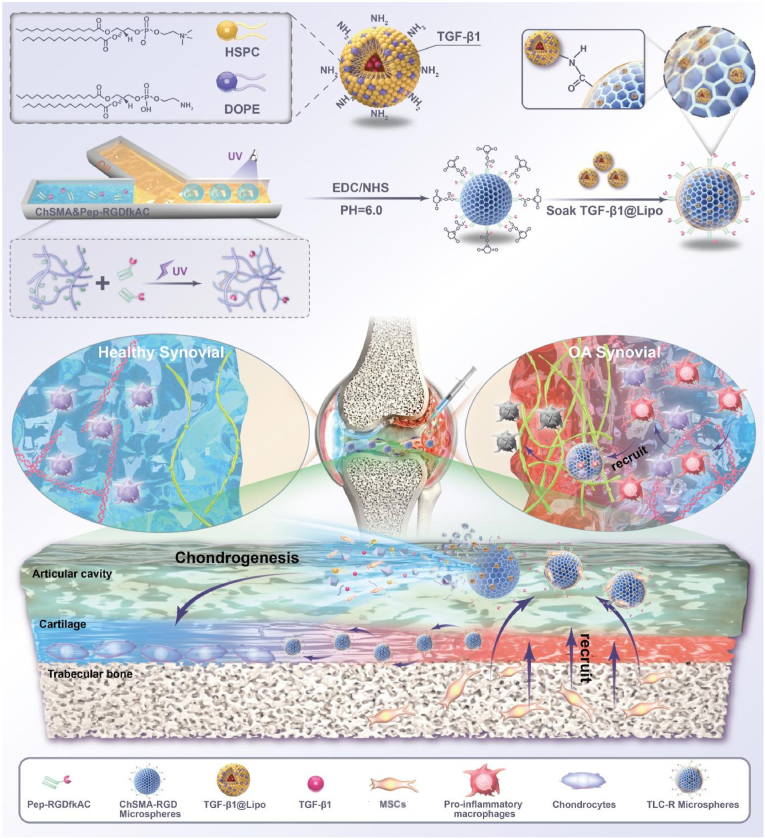
The manufacturing process protocol of TLC-R and its application in OA therapy. TLC-R is manufactured by a combination of microfluidic and chemical cross-linking techniques. It can treat OA by recruiting pro-inflammatory macrophages and MSCs, reducing inflammation, promoting chondrocyte differentiation, modulating chondrocyte metabolism, and improving lubrication. TLC-R is effective in the treatment of OA [162].

Recent advancements in hydrogel microspheres with anti-inflammatory, antioxidant, and immunomodulatory properties have shown promise in preventing secondary cartilage injury, facilitating functional repair, and enhancing regeneration. Xiao et al. developed hydrogel microspheres loaded with chemokines, macrophage antibodies, and engineered extracellular vesicles. These microspheres, prepared using microfluidic technology, efficiently captured and reprogrammed pro-inflammatory macrophages in the joint cavity, improving the inflammatory microenvironment and mitigating cartilage matrix degradation [134]. Similarly, Zhou et al. designed TGF-β1@Lipo@ChSMA-RGD microsphere (TLC-R) with cellular recruitment capabilities. These microspheres recruited macrophages and MSCs to the drug delivery site and released TGF-β1 and chondroitin sulfate to promote MSC chondrogenic differentiation and reprogram macrophages into an anti-inflammatory phenotype. This dual regulatory strategy supported cartilage regeneration and reduced inflammation that drives disease progression (Fig. 4) [162]. Oxidative stress is another critical factor in OA progression. It not only degrades the extracellular matrix of chondrocytes and compromises the structural and functional integrity of articular cartilage [168] but also disrupts the balance between anabolic and catabolic metabolism in joints, thereby accelerating cartilage degradation and impairing its repair and regeneration [169,170]. To address this, Cao et al. identified superoxide dismutase 3 (SOD3) as a key molecule for enhancing the antioxidant capacity of chondrocytes through single-cell sequencing analysis. They subsequently injected hydrogel microspheres enriched with SOD3 exosomes into the joint cavities of OA mice. The treatment altered the oxidative microenvironment, reduced oxidative stress, enhanced cartilage regeneration, and improved joint function, presenting a promising approach for future OA therapies [30]. Therefore, hydrogel microspheres enable precise immunomodulation through surface functionalization while simultaneously facilitating the spatiotemporal controlled release of antioxidants and pro-regenerative signals. This dual mechanism synergistically reverses the pathological microenvironment of osteoarthritis. Additionally, their injectable properties and multi-targeted intervention strategy overcome the limitations of traditional monotherapies, offering a novel approach for minimally invasive adaptive cartilage repair.

### 6.2 Regulation of chondrocyte metabolism

**Fig. 5:**
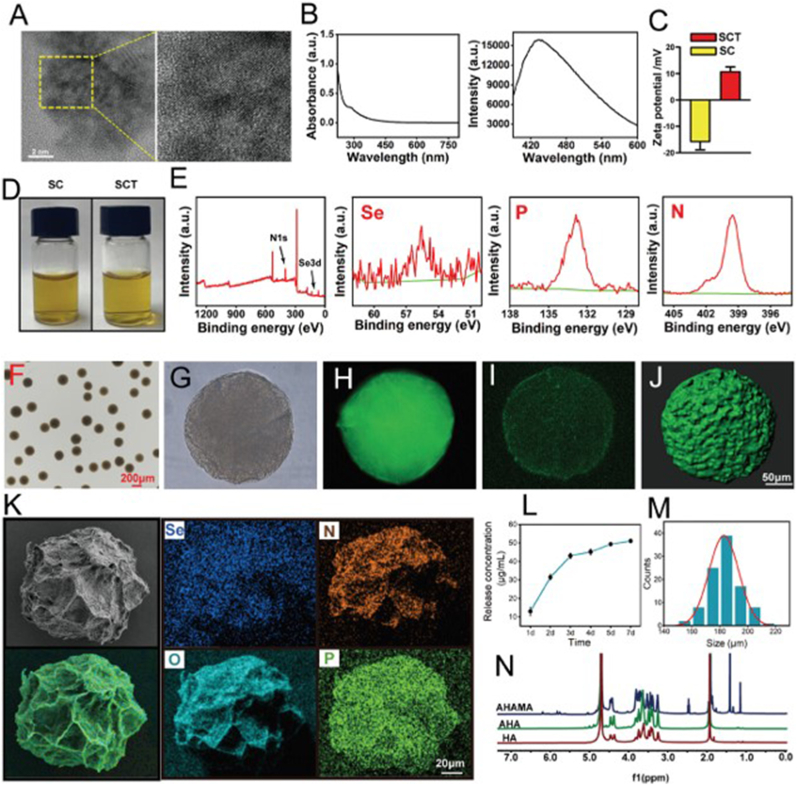
Characterization and preparation of highly permeable targeted bone/cartilage mitochondrial hydrogel microspheres. (A) TEM image of SCT. (B) UV absorption and fluorescence spectra of SCT. (C) Zeta potential measurements of SC and SCT in aqueous solution. (D) Images of SC and SCT in solution. (E) XPS spectra of elements Se, P, and N in SCT. (F) Optical microscope images of SCT-HA hydrogel microspheres prepared by microfluidic counting. (G) Optical microscope image of a single SCT-HA hydrogel microsphere (H) Fluorescence microscope image of a single SCT-HA hydrogel microsphere. (I) Laser confocal microscopy image of a single SCT-HA hydrogel microsphere. (J) 3D rendering of a single SCT-HA hydrogel microsphere. (K) SEM images of SCT-HA and elemental mapping. (L) Release profile of SCT-HA microspheres. (M) Particle size distribution of microspheres. (N) 1H NMR spectra of AHAMA, AHA, and HA were used to identify aldehyde and methacrylic anhydride groups [175].

During cartilage injury, chondrocyte metabolism undergoes significant alterations that impair their function and repair capacity [171]. In healthy cartilage, there is a balance between chondrocyte matrix synthesis and degradation. However, pathological factors disrupt this equilibrium by stimulating the release of matrix-degrading enzymes, leading to a dominance of matrix degradation over synthesis [172]. Additionally, elevated ROS triggered by oxidative stress further damages chondrocytes activates signaling pathways such as NF-κB, exacerbates inflammation, and promotes apoptosis [167,173]. Mitochondrial dysfunction also contributes to decreased ATP production, weakening cell viability and anabolic capacity, thereby limiting cartilage regeneration [174]. These metabolic imbalances exacerbate cartilage degradation and functional loss, underscoring the importance of regulating and optimizing chondrocyte metabolism for effective cartilage repair. Recent advancements in hydrogel microspheres have shown potential in addressing these challenges. Zuo et al. developed highly permeable micro/nano hydrogel microspheres encapsulated with selenium-doped carbon quantum dots (Fig. 5). This design overcame the spatial resistance of cartilage and precisely regulated matrix synthesis and catabolism, thereby promoting cartilage regeneration [175]. Chondroitin sulfate, a natural glycosaminoglycan and critical structural component of cartilage and other connective tissues, has also been explored for therapeutic applications [176]. Zhou et al. designed hydrogel microspheres loaded with chondroitin sulfate, gradually releasing the compound as the microspheres degraded. This sustained release promoted chondrocyte anabolism while inhibiting catabolic metabolism and inflammation over time [162]. In conclusion, hydrogel microspheres effectively repair mitochondrial dysfunction through the spatiotemporal antioxidant modulation of selenium-doped carbon quantum dots. Simultaneously, they enhance anabolism and continuously inhibit matrix-degrading enzyme activity via the sustained release of chondroitin sulfate. This dynamic regulation of anabolism and catabolism overcomes the limitations of traditional therapies, which intervene in metabolic disorders in a unidimensional manner.

### 6.3 Delivery platform

**Fig. 6:**
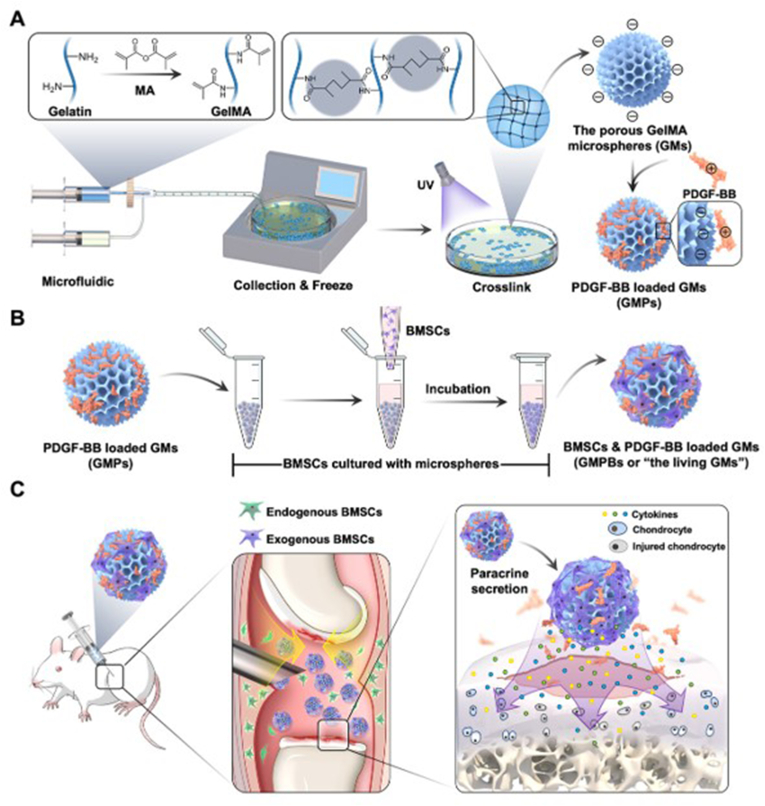
Schematic representation of *in vivo* hydrogel microspheres for regenerative repair of articular cartilage. (A) Hydrogel microspheres were prepared using microfluidics, and PDGF-BB was loaded onto the microspheres by electrostatic force to construct a GMP. (B) Construction of hydrogel microspheres loaded with BMSC and PDGF-BB by co-incubation with exogenous BMSC. (C) Enhanced paracrine properties of cells regulated by endogenous and exogenous regenerative mechanisms [25].

After cartilage injury, various growth factors, including transforming growth factor-β, insulin-like growth factor-1, fibroblast growth factor-18, and platelet-derived growth factor, are secreted to the injury site. These factors synergistically promote chondrocyte proliferation and differentiation, enhance matrix synthesis, and inhibit inflammatory responses, ultimately facilitating cartilage repair and regeneration [91,177,178]. Hydrogel microspheres carry therapeutic agents such as growth factors, drugs, bioactive substances, and cells. Their excellent biocompatibility, high porosity, and uniform, controllable size enhance tissue repair and modulate immune and inflammatory responses [179]. This integrated approach holds significant potential for cartilage regeneration and repair. Li et al. used freeze-drying microfluidic technology to create injectable porous hydrogel microspheres with long-lasting paracrine activity by incorporating platelet-derived growth factor-BB (PDGF-BB) and exogenous mesenchymal stem cells. These microspheres induced adhesion and proliferation of MSCs, enhanced extracellular matrix-cell interactions, and improved paracrine signaling. Additionally, they sustained the release of PDGF-BB, recruiting endogenous MSCs, prolonging paracrine activity, and effectively slowing the progression of OA (Fig. 6) [25]. Similarly, He et al. developed an injectable chondroitin sulfate hydrogel microsphere loaded with liposomes by covalently modifying photo-crosslinked methacryloyl, creating a dual-antioxidant drug delivery platform. In this system, liposome-loaded glycyrrhizin synergized with the degradation products of chondroitin sulfate to slowly and continuously scavenge reactive oxygen species via enzymatic degradation. This mechanism attenuated IL-1β-induced chondrocyte matrix degradation, inhibited M1 macrophage polarization, and suppressed inflammatory vesicle activation, thereby mitigating OA progression [29].

Studies have shown that certain drugs can promote cartilage repair; however, most small-molecule drugs have a short retention time in the joint cavity, leading to low delivery efficiency. Repeated administrations to counteract this limitation often increase the risk of adverse drug events and systemic side effects[[180], [181], [182]]. Tong et al. developed an injectable hydrogel microsphere system to address these challenges using microfluidic and photocrosslinking techniques. The system incorporated methacrylate-modified sulfonated azocup [4]aryl hydrocarbons (SAC4A-MA), methacrylate-acylated hyaluronic acid, and a matrix metalloproteinase-13 (MMP-13)-sensitive peptide. Owing to the hydrophobic deep cavity structure, phenolic units, and azo bonds of SAC4A-MA, these hydrogel microspheres exhibited high drug-loading capacity, ROS-scavenging ability, and hypoxia-responsive drug release. Under hypoxic and inflammatory microenvironments, the microspheres were degraded by excess MMP-13, specifically releasing the anti-inflammatory drug hydroxychloroquine. This process inhibited macrophage-induced inflammation, reduced oxidative stress, suppressed inflammatory factor expression, and prevented cartilage damage [28]. Similarly, Xia et al. designed dual-responsive hydrogel microspheres loaded with dihydromyricetin by anchoring gelatin methacrylate and benzene diboronic acid to hyaluronic acid methacrylate using microfluidic techniques. When injected *in vivo*, these microspheres effectively reduced cartilage wear and subchondral osteosclerosis. Furthermore, they promoted motor function recovery by restoring endogenous mitochondrial apoptosis and autophagy homeostasis [183].

An effective approach to treating cartilage injuries involves placing MSCs or chondrocytes into the injured area. However, this method faces significant challenges, such as low cell survival rates and difficulty in anchoring implanted cells, which limits its clinical application [184]. Researchers have explored hydrogel microspheres as cell delivery platforms to address these issues due to their unique properties and advantages, supporting cartilage repair and regeneration. In a study by Huang et al., injectable gelatin/glucosamine microspheres were prepared as scaffolds for cartilage cell delivery. *In vitro* experiments demonstrated that rabbit chondrocytes proliferated, migrated, and differentiated effectively within the microsphere scaffolds, with glucosamine promoting chondrocyte differentiation. *In vivo* experiments revealed that the scaffold exhibited excellent retention, biocompatibility, and suitable biodegradability. Histological and immunohistochemical analyses showed that the chondrocyte/frozen gel microsphere constructs formed ectopic functional cartilage tissue 21 days after subcutaneous implantation [185]. In another study, Cui et al. developed a novel 3D assembly technique to fabricate bioink microspheres containing allogeneic umbilical cord blood-derived mesenchymal stem cells (UCB-MSCs) using high-throughput microfluidics. This approach aimed to construct engineered osteochondral tissues with hierarchical structures. Results indicated that UCB-MSCs demonstrated robust chondrogenic and osteogenic differentiation within the bioink microspheres. Additionally, the assembled cartilage and bone modules fused seamlessly in the scaffolds, forming osteochondral tissues with hierarchical structures. This technique enables precise control over the spatial arrangement of cells and biomaterials, promotes tissue fusion and maturation, and offers a promising new strategy for osteochondral repair [186]. In summary, hydrogel microspheres synergistically promote chondrocyte anabolism while inhibiting catabolism by dynamically responding to the release of growth factors (e.g., TGF-β, PDGF) and antioxidants (e.g., dihydromyricetin). Simultaneously, their three-dimensional porous microenvironment enhances mesenchymal stem cell survival (>95 %) and chondrogenic differentiation. Additionally, the controlled release of therapeutic agents at targeted degradation sites enables localized, long-term cartilage repair, offering a comprehensive approach to restoring metabolic balance through an integrated “antioxidant-regeneration-structural reconstruction” strategy.

### 6.4 Improvement of lubrication

**Fig. 7:**
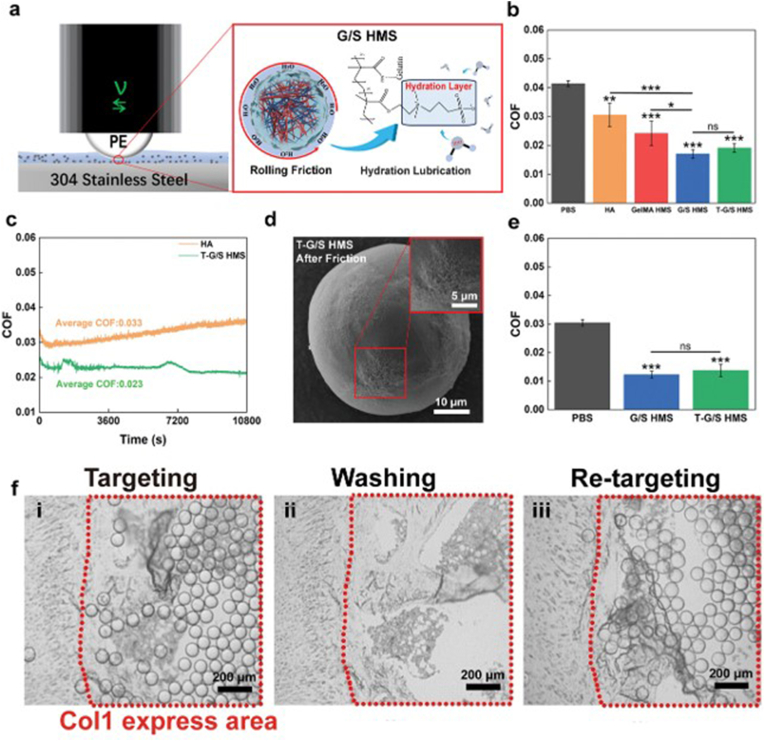
Lubrication performance testing of G/S hydrogel microspheres. (A) Schematic diagram of the tribological testing method and lubrication structure of G/S hydrogel microspheres (B) COF histograms of PBS, HA, GelMA HMS, G/S HMS, and T-G/S HMS (C) COF-time curves of T-G/S long-time fictitious test. (D) SEM images of T-G/S HMS after friction (E) COF histograms of PBS, G/S HMS, and T-G/S HMS when a Col1-coated 304 stainless steel plate was used as the friction partner. (F) T-G/S HMS aggregated on Col1 expression area of human facet joint section before and after washed out [126].

The depletion of synovial fluid in joints is closely linked to the development of age-related osteoarthritis, a condition that induces inflammation and cartilage degeneration [187]. Under normal physiological conditions, synovial fluid, composed of various biomolecules, serves as an effective lubricant, reducing inter-articular friction and mechanical stress. This lubrication protects cartilage from damage and provides a stable microenvironment for repair and regeneration [188]. However, with advancing age and the influence of pathological factors, synovial fluid concentrations decline, leading to increased joint wear and degeneration [189]. Thus, maintaining adequate intra-articular lubrication is essential for joint mobility, cartilage repair, and preventing degenerative changes. The hydrophilic polymer network structure of hydrogel microspheres, combined with their excellent mobility and flexibility, allows them to absorb and retain significant amounts of water. Unlike conventional hydrogels, hydrogel microspheres adapt to irregular joint spaces and accommodate joint motion, making them ideal for intra-articular applications [190,191]. Hou et al. developed amphoteric ionic lubricating hydrogel microspheres encapsulating metformin. These microspheres achieved enhanced lubricating properties and prolonged drug release through hydration and electrostatic interactions with metformin. *In vitro* studies demonstrated that the microspheres exhibited excellent lubricity, mechanical stability, and sustained drug release. They effectively regulated chondrocyte metabolic balance, improved the structure and metabolism of cartilage and subchondral bone, and mitigated cartilage senescence and OA progression [24]. He et al. designed antibody-mediated targeted hydrogel microspheres by anchoring an anti-type I collagen antibody (Anti-Col1) onto a biocompatible hydrogel microsphere matrix composed of gelatin methacrylate and poly (methacryloyl sulfobetaine) (PSBMA). These microspheres utilized the high hydrophilicity of PSBMA and the dynamic interaction between Anti-Col1 and collagen type I at injury sites to achieve precise lubrication. This targeted approach effectively alleviated symptoms, slowed disease progression, and highlighted the potential of targeted lubrication strategies in cartilage regenerative repair (Fig. 7) [126]. In summary, hydrogel microspheres facilitate cartilage metabolism repair through dynamic lubrication and targeted drug delivery while inhibiting inflammatory matrix degradation via the sustained release of therapeutic molecules (e.g., metformin). This dual-action approach supports the reconstruction of both the joint's mechanical microenvironment and metabolic homeostasis.

### 6.5 Recruitment of endogenous stem cells

Autologous stem cells, derived from the patient's own body, significantly reduce the risk of immune rejection and enhance treatment safety. These cells actively participate in the natural repair process and exhibit strong adaptability to the microenvironment at the injury site, offering the potential for long-lasting and stable therapeutic effects [184]. Consequently, stem cell therapy remains one of the most promising strategies for treating cartilage injuries [192]. However, ensuring effective delivery and stable retention of stem cells at the injury site remains a significant challenge. Addressing this issue, hydrogel microspheres have emerged as a novel platform for supporting cartilage repair by recruiting endogenous stem cells [193]. Zhou et al. developed multifunctional TLC-R hydrogel microspheres by incorporating liposomes containing TGF-β1 into chondroitin methacrylate sulfate microspheres modified with arginine-glycyl-aspartate peptide. These hydrogel microspheres effectively recruited BMSCs and macrophages, facilitated chondrogenic differentiation of BMSCs, improved cartilage metabolic homeostasis, and promoted cartilage regeneration [162]. Similarly, Dai et al. designed a functional injectable hydrogel system for in situ cartilage regeneration. This system, based on collagen embedded with microspheres, enabled the spatiotemporal sequential release of kartogenin, enhancing endogenous MSC recruitment and cartilage regeneration. This approach demonstrates significant potential in cartilage tissue regeneration [130]. In conclusion, hydrogel microspheres effectively recruit endogenous stem cells, promote cartilage differentiation, and inhibit catabolic enzyme activity through the spatiotemporal controlled release of stem cell homing signals (e.g., TGF-β1/KGN) and metabolic regulators (e.g., RGD peptide). This approach offers a novel strategy for adaptive metabolic homeostasis reconstruction in cartilage repair.

## 7 Conclusion and outlook

In recent years, hydrogel microspheres have gained significant attention for their applications in cartilage injury repair. This paper provides a detailed overview of cartilage repair strategies, including cartilage drilling and grinding, osteochondral grafting, cell transplantation, periosteal or chondral membrane grafting, and 3D scaffolding. Furthermore, we summarize the preparation methods of hydrogel microspheres, such as microfluidics, emulsification, electrospray, photolithography, and 3D printing. These techniques can be employed individually or in combination to produce multifunctional hydrogel microspheres, each offering distinct advantages and features. Researchers should carefully select and combine these methods based on specific experimental or clinical needs. In the context of cartilage repair, hydrogel microspheres perform various critical functions, including controlling inflammation, modulating immune responses, regulating chondrocyte metabolism, serving as delivery platforms, improving lubrication, and recruiting endogenous stem cells. This study aims to serve as a valuable reference for advancing research on hydrogel microspheres and their practical applications in cartilage injury repair.

Numerous studies have developed innovative hydrogel microspheres using various strategies to expand their applications in cartilage injury repair. However, several challenges and limitations remain that must be addressed in future research. Expanding the production scale of hydrogel microspheres remains challenging due to technical bottlenecks. Current laboratory preparation methods often fail to meet industrial production requirements, leading to issues such as poor batch-to-batch consistency, low production efficiency, and high costs. To address these challenges, future research should prioritize the development of efficient and standardized microsphere fabrication equipment, such as automated microfluidic-based systems, to enhance size uniformity, improve production efficiency, and reduce costs. Additionally, optimizing the cross-linking methods of hydrogel materials is essential to maintaining a stable three-dimensional structure and geometric properties during scale-up. This optimization will better replicate the microarchitecture of cartilage tissue, create a more suitable cellular growth environment, and lower production costs, ultimately facilitating clinical translation and application Second, further investigation is needed to elucidate the interaction mechanisms between hydrogel microspheres and chondrocytes to enhance biocompatibility, promote cell adhesion, proliferation, and differentiation, and accelerate cartilage tissue regeneration. Additionally, the long-term stability and safety of hydrogel microspheres *in vivo* must be thoroughly evaluated, including their degradation rate and the effects of their metabolites, to ensure safety during cartilage repair. Future research could also focus on integrating the advantages of different materials to develop multifunctional hydrogel microspheres through organic combinations and cross-linking modulation. Such microspheres should provide mechanical support, slow-release capabilities, and anti-inflammatory effects, creating a favorable microenvironment for cartilage repair. Moreover, combining hydrogel microspheres with other cartilage regeneration strategies, such as stem cell therapy and tissue engineering scaffolds, could harness complementary advantages and achieve synergistic effects, thereby improving the overall therapeutic outcomes in cartilage injury repair.

The application of hydrogel microspheres in cartilage injury repair holds significant promise. Advances in biotechnology and preparation techniques are expected to enable the design of personalized, multifunctional hydrogel microspheres with stable physicochemical properties, providing a strong foundation for their early clinical application.

## CRediT authorship contribution statement

**Zehua Wang:** Writing – original draft, Visualization. **Xiaoxia Li:** Writing – review & editing, Visualization. **Yaping Jiang:** Software, Investigation, Data curation. **Tingyu Wu:** Supervision, Conceptualization. **Sijia Guo:** Methodology, Investigation, Formal analysis. **Tao Li:** Writing – review & editing, Supervision, Project administration, Funding acquisition.

## Associated data

This section collects any data citations, or data availability statements included in this article.

## Declaration of competing interest

The authors declare that they have no known competing financial interests or personal relationships that could have appeared to influence the work reported in this paper.

## Data Availability

No data was used for the research described in the article.

## Abbreviations

OA: Osteoarthritis
ECM: Extracellular matrix
MSCs: Mesenchymal stem cells
BMSCs: Bone marrow mesenchymal stem cells
WPU: Waterborne polyurethane
SDF-1α: Stromal-derived factor-1α
TGF-β1: Transforming growth factor-β1
HA: Hyaluronic acid
PEG: Polyethylene glycol
PLGA: Polylactic-co-glycolic acid
GelMA: Methacrylate gelatin
RGD: Arginine-glycyl-aspartic acid
W/O: Water-in-oil
O/W: Oil-in-water
W/O/W: Water-in-oil-in-water
O/W/O: Oil-in-water-in-oil
ADSCs: Adipose-derived stem cells
Alg-Gel-ADSCs MSs: Alginate-gelatin hydrogel microspheres loaded with adipose-derived stem cells
CAD: Computer-aided design
GEF: Gefitinib
G-CSF: Granulocyte colony-stimulating factor
SRM: Slow-release microspheres
IL-1β: Interleukin-1 β
MMPs: Matrix Metalloproteinases
ROS: Reactive oxygen species
TLC-R: TGF-β1@Lipo@ChSMA-RGD microsphere
SOD3: Superoxide dismutase 3
PDGF-BB: Platelet-derived growth factor-BB
SAC4A-MA: Sulfonated azocup [4]aryl hydrocarbons
MMP-13: Matrix metalloproteinase-13
UCB-MSCs: Umbilical cord blood-derived mesenchymal stem cells
Anti-Col1: Anti-type I collagen antibody
PSBMA: Poly (methacryloyl sulfobetaine)
